# Crosstalk between Tryptophan Metabolism and Cardiovascular Disease, Mechanisms, and Therapeutic Implications

**DOI:** 10.1155/2017/1602074

**Published:** 2017-03-09

**Authors:** Gang Liu, Shuai Chen, Jin Zhong, Kunling Teng, Yulong Yin

**Affiliations:** ^1^Key Laboratory of Agro-Ecological Processes in Subtropical Region, Institute of Subtropical Agriculture, Chinese Academy of Sciences, National Engineering Laboratory for Pollution Control and Waste Utilization in Livestock and Poultry Production, Hunan Provincial Engineering Research Center of Healthy Livestock, Scientific Observing and Experimental Station of Animal Nutrition and Feed Science in South-Central, Ministry of Agriculture, Hunan Co-Innovation Center of Animal Production Safety, Hunan 410125, China; ^2^State Key Laboratory of Microbial Resources, Institute of Microbiology, Chinese Academy of Sciences, Beijing 100101, China; ^3^University of Chinese Academy of Sciences, Beijing 100049, China; ^4^Laboratory of Animal Nutrition and Human Health, School of Biology, Hunan Normal University, Changsha, Hunan, China; ^5^College of Animal Science, South China Agricultural University, Guangzhou 510642, China

## Abstract

The cardiovascular diseases (CVD) associated with the highest rates of morbidity are coronary heart disease and stroke, and the primary etiological factor leading to these conditions is atherosclerosis. This long-lasting inflammatory disease, characterized by how it affects the artery wall, results from maladaptive immune responses linked to the vessel wall. Tryptophan (Trp) is oxidized in a constitutive manner by tryptophan 2,3-dioxygenase in liver cells, and for alternative cell types, it is catalyzed in the presence of a differently inducible indoleamine 2,3-dioxygenase (IDO1) in the context of a specific pathophysiological environment. Resultantly, this leads to a rise in the production of kynurenine (Kyn) metabolites. Inflammation in the preliminary stages of atherosclerosis has a significant impact on IDO1, and IDO1 and the IDO1-associated pathway constitute critical mediating agents associated with the immunoinflammatory responses that characterize advanced atherosclerosis. The purpose of this review is to survey the recent literature addressing the kynurenine pathway of tryptophan degradation in CVD, and the author will direct attention to the function performed by IDO1-mediated tryptophan metabolism.

## 1. Introduction

Tryptophan (Trp), an essential amino acid, constitutes a central component in human and animal protein synthesis, and it serves as the sole source of substrates that facilitate the generation of a range of crucial molecules. Trp precedes and indicates the synthesis of proteins, nicotinamide adenine dinucleotide (NAD), nicotinic acid, and serotonin (namely, the neurotransmitter) [1, 2]. For mammalian species, the kynurenine (Kyn) pathway is Trp's central catabolic route, featured in 95% of peripheral Trp metabolism in mammals; furthermore, it results in NAD's biosynthesis, as NAD functions as a crucial cofactor [3].

The highest rates of global morbidity are associated with cardiovascular disease (CVD), and atherosclerosis is the primary etiological factor leading to various manifestations of CVD, including coronary heart disease and stroke [4]. One of the critical factors in CVD pathogenesis is the immune response, and a clinical solution remains to be identified [5, 6]. Atherosclerosis occurs due to the manner in which low-density lipoprotein (LDL) accumulates and is retained in the arterial wall, and this leads to maladaptive responses from T cells and macrophages [7]. Scholars in recent years have directed significant energy towards the examination of the Kyn pathway and the role it plays in CVD pathogenesis, and because several hypotheses have suggested that various factors, including oxidative stress, immune activation, and inflammation, are central to the pathogenesis of atherosclerosis and CVD, a critical area of future investigation is to examine to potential part played by the Kyn pathway in CVD regarding these factors.

## 2. Tryptophan Metabolism and the Kynurenine Pathway

**Figure 1:**
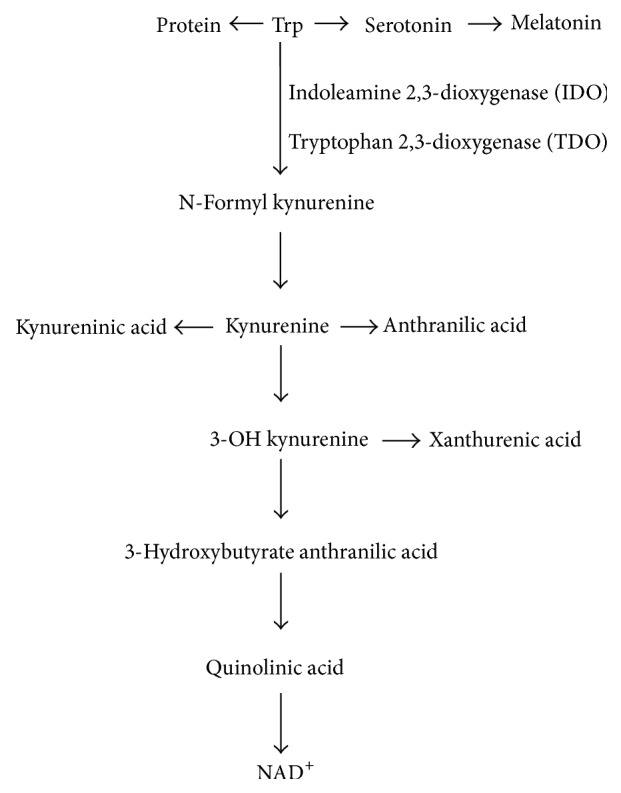
Schematic illustration of Trp catabolism along the mammalian Kyn pathway.

Trp hydroxylase facilitates the biotransformation of approximately 5% Trp via metabolism to 5-hydroxy Trp, and this generates serotonin by decarboxylase (an amino acid). Lastly, through* N*-acetyltransferase, serotonin is metabolized to melatonin. Via the Kyn pathway, the degradation of the other 95% of Trp is converted to kynurenine, and the regulation of this primarily occurs with a pair of rate-limiting enzymes, tryptophan 2,3-dioxygenase (TDO) and indoleamine 2,3-dioxygenase (IDO1). Each of these enzymes incorporates one noncovalently bound iron-protoporphyrin IX to every monomer, and TDO and IDO1 are members of the oxidoreductase family. Specifically, the enzymes are associated with the family of oxidoreductases that act on single donors with O_2_ as the oxidant and the inclusion of two oxygen atoms into the substrate (oxygenases) [8, 9]. The expression of IDO1 occurs at basal levels as a consequence of antigen-presenting cells, including macrophages and dendritic cells, and this procedure is driven to a significant extent by IFN-*γ*, the proinflammatory cytokine, and type I interferons, tumor necrosis factor, and lipopolysaccharide (LPS) (the latter three to a less significant degree) [10]. Considerable scholarly attention has been directed towards the immunoregulatory function played by Trp metabolism in the immune system, and most studies have centered on the role of IDO1; this rate-limiting enzyme governs the rate-limiting step of Trp catabolism. Kynureninase, after it has been synthesized by IDO1, uses Kyn to generate anthranilic acid (AA) [11]. Additional steps in the Kyn pathway relate to the degradation of kynurenine to the sequential production of 3-hydroxybutyrate kynurenine and 3-hydroxybutyrate anthranilic acid (3-HAA) or xanthurenic acid in the presence of kynurenine-3-monooxygenase (KMO) and kynureninase or kynurenine aminotransferase, respectively. 3-HAA is further metabolized to quinolinic acid (QA), the excitotoxin, which is a potent convulsant and excitant [12]. Furthermore, studies have demonstrated that kynurenine aminotransferase metabolizes Kyn to generate kynurenic acid (KYNA) [13]. Due to its N-methyl-D-aspartate (NMDA) receptor antagonist characteristics, KYNA is a neuroprotective compound [12]. The manner in which KMO is expressed and acts is improved by IFN-*γ* in the context of human macrophages and microglia cells [14], and an increase in KMO expression is linked to significant levels of TNF-*α* and IL-6 in the brains of rats after a systemic inflammatory challenge [15].  Figure 1 provides a schematic illustration of the ways in which the critical enzymes and substrates linked to the Trp metabolic pathway are associated with one another [9], and it also demonstrates the primary immune-related active substances, including kynurenine, quinolinic acid, 5-hydroxytryptamine (5-HT), and melatonin.

Preliminary research in this area mainly attributed the Kyn pathway with a central function in the generation of nicotinic acid or vitamin B3 [16]. Nevertheless, after the observation that modifications of Trp metabolism are present in numerous central nervous system conditions, attention moved towards the produced enzymes and metabolites, subsequently denoted as kynurenines. One of the critical findings was that QA operates as a potent convulsant and excitant [12] and, as such, resulted in convulsive responses when inserted into mouse brain ventricles. Furthermore, researchers found that QA functions as a selective NMDA receptor agonist [17]. AS, Trp, Kyn, AA, 3-hydroxybutyrate kynurenine, and xanthurenic acid readily cross the blood-brain barrier [18, 19]. The impacts that systemic Trp has on the brain Kyn pathway is partly facilitated by its peripheral conversion to Kyn and 3-OHkyn. An additional driver ensures entry of these metabolites into the brain. Kynurenic acid, 3-HAA, and QA, primarily as a consequence of the polar nature and the seeming absence of effective transition procedures, are not the same as a range of different kynurenine pathway metabolites because they cannot effectively cross the blood-brain barrier [18]. Therefore, their formation occurs in a local manner inside the brain.

## 3. Kynurenine Pathway and Immune Responses

Research has identified that a key function of the Kyn pathway relates to the pathological regulation of the innate and adaptive immune system [3]. In a prospective multicenter study involving a 986-person sample group, comprised entirely of individuals in the young adult age range, investigators noted that the activity of IDO1 is significantly associated with carotid artery intima-media thickness (IMT) in females. Specifically, IDO1 activity displayed a significant association with a range of atherosclerosis risk factors for the female population, including age, LDL cholesterol (LDL-C), and BMI. Moreover, IFN-*γ* was identified as the primary IDO1 inducer in vitro and in vivo, and the presence of IFN-*γ* facilitated an increase in intracellular IDO1 transcription [20, 21]. Another study identified alternative inflammatory factors as less prominent inducers of IDO1, including IFN-*α*, IFN-*β*, LPS, and cytotoxic T lymphocyte-associated antigen-4 [22].

Contemporary research findings have contributed to a body of knowledge in which a minimum of three mechanisms that facilitate the initiation of immunological suppression are understood. It is notable that all of the identified immunosuppressive impacts are aligned with IDO1 activation and its downstream effects on specific groups of T cells. Initially, active IDO1 facilitates the depletion of Trp in local tissue microenvironments and, in turn, it drives the promotion of metabolite generation associated with the Kyn pathway. After IDO1 induction, which results in the inhibition of the propagation of reactive T lymphocytes, Trp levels are depleted, which increases the degree to which T lymphocytes are susceptible to cell death [23]. In vitro Trp depletion leads to cell cycle impedance of activated T cells, and this similarly increases the likelihood of cell death [24].

Second, the subsequent rise in kynurenine metabolites, including Kyn, QA, and 3-HAA, impedes propagation and, following this, facilitates the initiation of selective cell death regarding T helper 1 (TH1) lymphocytes, which respond to antigen-presenting cells [25]. Research findings have demonstrated that kynurenine results in negative impacts regarding several phenomena, including how immune responses are regulated, the inhibition of T cell and NK cell propagation, and the regulation of immunogenic dendritic cells [26]. In the context of inflammation conditions, Trp degrades quickly to QA, and QA and 3-hydroxybutyrate kynurenine have the potential to induce the selective cell death in vitro of TH1 and not TH2 cells. Therefore, by suppressing and removing T lymphocytes, Trp metabolism has the potential to impact immunity [27].

Third, it is important to recognize that an increase in the frequency of regulatory T cells positive for forkhead box P3 (FOXP3+) via TGF*β* induction occurs when two conditions, namely, the presence of kynurenine metabolites and Trp depletion, are met, and the simultaneous presence of these conditions also heightens the effect on naïve T cells [27]. This effect significantly advances immune tolerance and a negatively formulated feedback loop, thereby driving immune response regulation [28].

## 4. Kynurenine Pathway and Cardiovascular Disease

The overexpression of IDO1 accompanied by increased Trp catabolism has been shown to stem from chronic systemic low-grade inflammation (CSLGI), which is a predictive factor for the results of CVD. The enhanced degradation of Trp was linked to inflammation in [29] by an observation of the increased plasma Kyn to Trp ratio (Kyn/Trp) (KTR). Here, a sample group of mature participants from Finland demonstrated that the aforementioned ratio is positively associated with BMI, LDL, triglycerides, and waist circumference while it is negatively associated with high-density lipoprotein (HDL) [30]. Studies were conducted on an expansive cohort taking a broad sample of all demographic groups, and it was noted that IDO1 activity (via the KTR) was positively associated with the preliminary stages of atherosclerosis and increased carotid artery IMT for males and females; this finding indicates that IDO1 constitutes a viable indicator of atherosclerosis [31]. Increased IDO1 expression was identified in the macrophage-loaded core of atherosclerotic plaques in human participants [32], and another study demonstrated that low Trp plasma concentration and a high KTR are characteristic of individuals suffering from coronary heart disease [33]. Moreover, a high KTR is a sensitive indicator of severe coronary events for individuals displaying no history of coronary artery disease [34]. Therefore, KTR can be used to forecast critical coronary events and is also useful in determining all-cause mortality for individuals suffering from coronary artery disease [35]. A relationship was observed between KTR and IMT for individuals suffering from hemodialysis while being classified as high risk for CVD [36], and it was reported that increased Trp degradation is associated with neopterin plasma concentrations [35]. This finding constitutes a biomarker of cell-mediated immune activation and is connected to atherosclerotic CVD [37]. Epidemiological research indicates that the Kyn pathway's activity, as manifested in the plasma Kyn/Trp, is associated with the stroke-induced inflammatory response, the degree of which strokes is severe, and chronic clinical results [38]. Kyn, which has been shown to precede and indicate KYNA, was identified as considerably reducing neuronal damage and infarct volume, and this was determined in a study involving the preischemic intraperitoneal administration in various rat models of brain ischemia-hypoxia [39]. Furthermore, 3-hydroxykynurenine, as is the case with the KTR, has been linked to the appearance of CVD in individuals suffering from chronic renal disease, and this was verified in an independent manner [40].

In preliminary investigations, 3-HAA was frequently denoted as a Trp metabolite resulting in antioxidant and anti-inflammatory impacts. Research has shown that 3-HAA in mitochondrial mechanisms impedes oxygen uptake by mitochondrial respiration with NAD-dependent substrates, the uncoupling of the respiratory chain, and oxidative phosphorylation [41]. In addition, various studies have examined the ways in which 3-HAA results in cell death, by associating it with the apoptosis induction in monocyte/macrophage cell lines [42], identifying a link between 3-HAA and apoptosis in activated T cells [43], and demonstrating that 3-HAA facilitates the inhibition of nuclear factor-*κ*B activation [44]. Experimental findings indicate that 3-HAA has a significant function regarding atheroprotection in that it facilitates the regulation of lipoprotein metabolism.* LDLr*^−/−^ and* IDO*^−/−^ double knockout mice displayed a considerable increase in serum lipids, especially triglycerides [45]. Furthermore, the administration of 3-HAA to LDL receptor knockout (*LDLr*^−/−^) mice facilitated a significant decrease in overall plasma cholesterol and triglyceride levels, and the former effect was attributed to the lower chylomicron/VLDL ratio. In addition, 3-HAA led to a significant increase in HDL-C [46].

## 5. Therapeutic Implications and Concluding Remarks

CSLGI linked to conventional CVD risk factors leads to an increase in Trp degradation. Therefore, one of the critical objectives in developing appropriate therapies for the symptoms of CVD patients is to normalize Trp metabolism. Practitioners should be aware that a high KTR may indicate a natural immune reaction, meant to combat inflammation and, furthermore, that the Kyn pathway can modulate vascular inflammation and atherosclerosis in a direct or indirect manner. Considering that IDO1 inhibition facilitates a reversal in septic shock associated-hypotension and, moreover, diminishes the likelihood of fatality, Kyn constitutes a fruitful area of investigation regarding the creation of therapies for hypertension.

More research is needed to gain comprehensive insight into the function of the Kyn pathway in the modulation of cardiovascular risk factors, atherosclerosis, and vascular inflammation. Furthermore, the nature of the Kyn pathway's related and initiated molecular mechanisms of action require more in-depth research. Specifically, future studies should focus on an investigation of the degree to which these parameters constitute the potential foundation of accurate and effective biologically informed therapies that can be implemented to heighten the likelihood of patient recovery from CVD.
